# Pan-cancer signaling landscape linked to endothelial and immune Sphingosine-1-phosphate receptor 1 (S1PR1) expression

**DOI:** 10.1007/s40203-026-00676-7

**Published:** 2026-06-20

**Authors:** Yazmin Torres-Santos, Yarely Mabell Beltrán-Navarro, Guadalupe Reyes-Cruz, José Vázquez-Prado

**Affiliations:** 1 Department of Pharmacology, Centro de Investigación y de Estudios Avanzados del Instituto Politécnico Nacional (Cinvestav), Av. Instituto Politécnico Nacional 2508, Col. San Pedro Zacatenco, 07360 Apartado Postal 14-740, 07000 Mexico City, Mexico; 2 Department of Cell Biology, Centro de Investigación y de Estudios Avanzados del Instituto Politécnico Nacional (Cinvestav), Av. Instituto Politécnico Nacional 2508, Col. San Pedro Zacatenco, 07360 Apartado Postal 14-740, Mexico City, Mexico

**Keywords:** Endothelial signaling, Immune signaling, RhoGEFs, Tumor microenvironment, Endothelial phosphoproteome, Immune phosphoproteome

## Abstract

Sphingosine-1-phosphate (S1P) promotes tumor growth and dissemination. Chronic positive feedback communication circuits between cancer and stromal cells involve S1P type-1-receptor (S1PR1) activating cell-type specific signaling networks. We hypothesized that such cell-type specific signaling components would be identifiable by rational, unbiased analysis of public oncogenomic and phosphoproteomic datasets. Guided by S1PR1 expression, we used data mining strategies applied to 32 cancer type datasets of the TCGA oncogenomics program, aiming to identify pan-cancer endothelial and immune S1PR1 signaling partners statistically correlated with patient survival. Gene ontology analysis and unbiased clustering of endothelial and immune S1PR1-signaling partners were used to reveal cell type-specific signaling components that individually and grouped, as transcriptional signatures, were statistically linked to patient survival. Furthermore, the breast cancer CPTAC dataset was analyzed focusing on the signaling phosphoproteome linked to S1PR1 expression. Oncogenic S1PR1 signaling companions included endothelial regulators of cell migration such as Ephexin5, a RhoGEF encoded by the ARHGEF15 gene, and RhoJ, a small Rho GTPase. The immune signaling repertoire linked to S1PR1 expression and patient survival included DOCK2, Vav1 and Rac2. Among the S1PR1 phospho-signaling partners, endothelial ARHGAP24, ARHGAP6, ARHGAP31, and TNS1, and immune ARHGAP25, known to be involved in cytoskeletal reorganization and cell mobilization, clustered as phosphoproteins within a subgroup of breast cancer patients. Given the pharmacological relevance of S1PR1 in endothelial and immune settings, revealing the identity of signaling molecules linked to S1PR1 expression provides useful information to further investigate therapeutic strategies targeting these pathways in the vascular and immune systems.

## Introduction

Sphingosine-1-phosphate (S1P) is a physiological bioactive lipid agonist that plays fundamental roles controlling endothelial and immune processes such as maintenance of vascular integrity and immune cell trafficking, by stabilizing endothelial cell adhesions and controlling egress of T lymphocytes from lymph nodes, respectively (Cartier and Hla 2019; Hallisey and Schwab 2023; Weigel et al. 2023). These processes involve multiple signaling effectors in charge of spatiotemporal regulation of cell adhesion, migration, and survival, mainly elicited by type 1 S1P receptors (S1PR1). Canonical signaling by S1PR1 occurs through the Gi family of heterotrimeric GTPases (Anwar and Mehta 2020; Reinhard et al. 2017), activating Gβγ-dependent effectors linked to cell migration such as phosphatidylinositol 3-kinase, beta and gamma isoforms (Guzman-Hernandez et al. 2009; Igarashi and Michel 2001), as well as various guanine nucleotide exchange factors, promoting cytoskeletal reorganization upon Rac1 activation (Kajimoto et al. 2018; Li et al. 2005; Singleton et al. 2005). S1PR1 interacts with P-Rex1 (Ledezma-Sanchez et al. 2010), an essential multidomain guanine nucleotide exchange factor that directly activates Rac1 (Hampson et al. 2021; Wang et al. 2017), a small GTPase of the Rho family (Welch et al. 2002). S1P-dependent migratory response is enhanced by P-Rex1, which contributes to maintaining S1PR1 at the plasma membrane (Baker et al. 2025; Ledezma-Sanchez et al. 2010), thereby linking chemotactic signaling to cell migration.

Oncogenic S1PR1 signaling has been related to tumor-induced angiogenesis, inflammation, invasion, metastasis and chemoresistance. Given the contribution of the vascular and immune systems in cancer development in which S1PR1 plays a documented role, targeting this receptor has been the focus of various preclinical studies (Deng et al. 2012; Lee et al. 2010; Nagahashi et al. 2018; Tao et al. 2023a, b). Chronic stimulation of S1PR1, maintained by cross communication between cancer and stromal cells, promotes tumor growth and metastatic dissemination (Lee et al. 2010; Lin et al. 2019; Nagahashi and Miyoshi 2024; Nagahashi et al. 2018), involving the recruitment of effector immune cells (Lee et al. 2010; Olesch et al. 2022). For instance, increased expression of S1PR1 in CD8+ T cells, residing in the skull’s bone marrow, enhances their infiltration to glioblastoma tumors (Dobersalske et al. 2024). S1PR1 in macrophages promotes cancer cell survival, therapeutic resistance and suppression of antitumor immunity, which converge on metastatic dissemination (Weichand et al. 2017). Furthermore, the S1PR1-STAT3 signaling axis in myeloid cells allow them to invade, proliferate, and resist apoptosis at premetastatic sites contributing to the arrival and metastatic establishment of cancer cells (Deng et al. 2012). Regarding vascular effects, S1PR1 either promotes tumor-induced angiogenesis or tumor vessel stabilization (Balaji Ragunathrao et al. 2019; Cartier et al. 2020). These contrasting processes differentially affect tumor growth (Balaji Ragunathrao et al. 2019; Cartier et al. 2020), depending on the molecular landscape linked to S1PR1 signaling (Balaji Ragunathrao et al. 2019). In murine models, during VEGF-dependent tumor-induced angiogenesis, the mechanism by which S1P-S1PR1 signaling amplifies the VEGF-VEGFR2 transduction pathway involves c-Abl1, a tyrosine kinase that phosphorylates VEGFR2, causing its retention at the plasma membrane and promoting Rac-dependent endothelial cell migration (Balaji Ragunathrao et al. 2019). Intriguingly, endothelial S1PR1 knockout increases tumor induced angiogenesis, indicating that S1PR1 stabilizes endothelial junctions (Cartier et al. 2020).

Given that the physiological roles of S1PR1 maintaining vascular stability and controlling homing and trafficking of lymphocytes are hijacked by growing tumors (Cartier and Hla 2019; Hallisey and Schwab 2023), characterization of the signaling repertoire linked to S1PR1 expression in tumors raises therapeutic implications considering that it might affect immune cell recruitment, promote tumor-induced angiogenesis or contribute to normalize the tumor vasculature, which would improve therapeutic strategies, depending on whether VEGFR2 is coexpressed (Balaji Ragunathrao et al. 2019; Cartier et al. 2020). Pharmacological implications of targeting S1PR1 include optimization of chemotherapeutic treatments (Lifshitz et al. 2017; Marmonti et al. 2020; Tao 2023a, b) and immune modulators (Olesch et al. 2022). Recently, S1PR1 expression has been statistically linked to tumor progression and patient survival. The published analysis included receptor mutations, posttranslational modifications and correlation with immune infiltration in different cancer types (Xiong et al. 2025; Zhong et al. 2020). These findings are consistent with the possibility that S1PR1 may be part of an oncogenic signaling repertoire linked to tumor progression and treatment effects. To further explore these possibilities, here we analyzed the S1PR1 pan-cancer signaling repertoire, based on coexpression data, particularly linked to immune and endothelial tumor stroma and its potential association with patient survival. Guided by S1PR1 expression in 32 cancer types of the TCGA program, we aimed to identify the pan-cancer repertoire of S1PR1 signaling companions. In addition, to reveal S1PR1-linked signaling phosphoproteome we analyzed the breast cancer CPTAC dataset (Krug et al. 2020). Since the signaling pathways activated by S1PR1 must be composed by a repertoire of transducers, enzymatic effectors, scaffolds and receptors coexpressed with S1PR1, those coexpressed throughout multiple cancer types are potentially linked to the role played by S1PR1 in cancer progression. Thus, clustered coexpression and gene enrichment analysis of signaling transcripts represent a consistent unbiased opportunity to identify S1PR1-linked pathways in multiple cancer types and set the basis to guide future hypothesis-driven questions aimed to test whether the signaling companions identified by coexpression analysis are indeed mechanistically linked to S1PR1 signaling in the tumor microenvironment.

## Materials and methods

### Cancer datasets

Thirty-two non-redundant cancer genomic and transcriptome TCGA datasets (Sanchez-Vega et al. 2018) were analyzed in the cBioPortal platform (Gao et al. 2013) (initial access date: October 2, 2024). Data included mRNA expression (RSEM, (HiSeq_RNAs Ilumina, normalized). Transcripts of signaling proteins coexpressed with S1PR1 (named as S1PR1 signaling companions, S1PR1-SC) in TCGA studies were identified from S1PR1-coexpression data. S1PR1 coexpression datasets for each cancer type were downloaded from cBioPortal. All the available transcriptomic information from 10,953 patients was included in the analysis, essentially done as previously described (Beltran-Navarro et al. 2022; Juan-Guadarrama et al. 2023). Transcripts coding for signaling proteins were identified and selected by tagging the data with a list of genes coding for proteins having structural characteristics consistent with their participation in signaling pathways, such as the presence of kinase, phosphatase, RhoGEF, RhoGAP, among other signaling domains as defined by the SMART platform (https://smart.embl.de/) (Letunic and Bork 2025). S1PR1-SC were defined as those with a Spearman correlation value of ≥ 0.3. S1PR1-SC were classified based on their structural characteristics as ligands, receptors, and components of the intracellular signaling hardware (including kinases, phosphatases, GTPases, guanine nucleotide exchange factors, GTPase activating proteins, signaling adaptors, among others), as shown in the respective figures. S1PR1-SC were organized in descending order of their Spearman’s correlation values before analyzing them as clustered signaling signatures using the complete Euclidean differences algorithm integrated in the analytical options of the Morpheus platform. Phosphoproteomic breast cancer datasets from the CPTAC study (Krug et al. 2020) were analyzed in the cBioPortal and Morpheus (https://software.broadinstitute.org/morpheus/ platforms, accessed December 3, 2025). All 122 patients were segregated (50:50) based on S1PR1 expression z-score mRNA. Phosphosites linked to endothelial or immune phosphoproteomes were identified by coexpression of proteins with PECAM1 and PTPRC with a Spearman’s correlation value of at least (≥ 0.3) restricting, in the case to endothelial phosphosites, to the top quartile. The corresponding phospho-signaling proteins were tagged with the list obtained from InterPro and clustered by Euclidian complete differences in the Morpheus platform.

### Clustered pan-cancer S1PR1-SC and enriched signaling pathways

S1PR1-SC correlation data were displayed in heatmaps initially arranged according to the corresponding Spearman’s correlation values starting with the highest values at the top left of the heatmap. Subsequently, hierarchical cluster analysis was done in the Morpheus platform (https://software.broadinstitute.org/morpheus) to group S1PR1-SC according to their similarity or differences, using appropriate algorithms such as One Minus Pearson for similarity and Complete Euclidean Difference for dissimilarity. Rows represent signaling transcripts, identified by the coding gene, as expressed in different cancer types, whereas columns represent the expression profile of S1PR1-SC of each cancer type. Transcriptional signatures defining Pan-cancer S1PR1-SC were integrated based on the identification of S1PR1-SC included in the main cluster containing at least two thirds of the cancer studies. In contrast, Cancer-Specific S1PR1-SC were identified sorting each list from higher to lower Spearman’s correlation values. Those transcripts with at least 0.3 Spearman’s correlation value were used to identify gene-set enrichments at the Metascape platform looking for Pan-cancer signaling pathways as classified by the Kyoto Encyclopedia of Genes and Genomes, KEGG database (https://metascape.org) (Zhou et al. 2019). Cancer-specific KEGG pathways were clustered using the Morpheus platform https://software.broadinstitute.org/morpheus/by the Euclidian distance metric and the complete linkage method algorithm based on the significant statistical values resulting from the gene enrichment analysis done at the Metascape platform.

### Endothelial and immune signaling landscape linked to S1PR1 expression

Non-overlapping S1PR1-SC groups were identified by selecting genes assigned exclusively to either endothelial or immune processes based on GO criteria (https://amigo.geneontology.org/amigo/landing database; GO endothelial (0001944) and GO immune (0002376)), followed by selection of those involved in cell migration and cytoskeletal dynamics (as indicated in the corresponding figures). These non-overlapping groups were further analyzed by coexpression with standard endothelial and immune markers. Only genes included in endothelial or immune pan-cancer clusters, analyzed with the Euclidian distance metric and the complete linkage method algorithm in the Morpheus platform, were retained for further analysis. Endothelial and immune pan-cancer signaling transcriptomes were identified by coexpression analysis with cell type-specific markers: PECAM1, coding for Platelet Endothelial Cell Adhesion Molecule-1 (also known as CD31) for endothelial cells and PTPRC, coding for the Protein Tyrosine Phosphatase Receptor Type C (also known as CD45), for lymphoid cells. Endothelial and immune coexpression lists were downloaded from the cBioPortal platform (https://www.cbioportal.org/). Gene enrichment analyses was done with the pan-cancer S1PR1-SC, pan-cancer endothelial S1PR1-SC, and pan-cancer immune S1PR1-SC to identify significant KEGG pathways. In addition, in the case of S1PR1, its coexpression with markers of epithelial, myeloid and fibroblast cells, to cover different cells of the tumor microenvironment, were included in the analysis using the following markers: FAP (Fibroblast Activation Protein, marker of fibroblasts), ITGAM (Integrin Subunit Alpha M, also known as CD11b, marker of myeloid cells) and EPCAM (Epithelial Cell Adhesion Molecule, marker of epithelial cells). S1PR1 correlation with each marker was illustrated in a heatmap showing its expression in different cells of the tumor microenvironment of 32 cancer types. Coexpression of PECAM1 or PTPRC mRNAs (RSEM, (HiSeq_RNAs Ilumina, normalized) with S1PR1-SC in the 32 TCGA cancer types was investigated in the cBioPortal platform (https://www.cbioportal.org/; initial accession date July 17, 2024).

### S1PR1-linked transcriptional signaling signatures statistically correlated with patient survival

Transcriptional signatures were identified from S1PR1-SC whose individual expression was statistically correlated with shorter patient survival. Individual survival curves were obtained organizing the groups of patients according to the “auto-select best cutoff” at the Kmplot platform (https://kmplot.com/analysis/) and analyzed using Cox regression algorithm with the tools available in the platform (Lanczky and Gyorffy 2021). This procedure was applied for each type of cancer and the identified pan-cancer signaling partners. The accumulated risk of the transcriptional signatures (S1PR1 and signaling companions altogether) was evaluated in KM plotter (https://kmplot.com/analysis/) in the “Custom” section, with a multivariate Cox regression analysis, dividing patients by the median expression of the collective genes per cancer study.

### Statistical analysis

Spearman’s correlation values were calculated in the cBioportal platform. Individual and multivariate Cox regression analyses were conducted in KM plotter for the cumulative risk signatures and verified in GraphPad Prism (6.01).

## Results

### Pan-cancer signaling landscape linked to S1PR1 expression

**Fig. 1:**
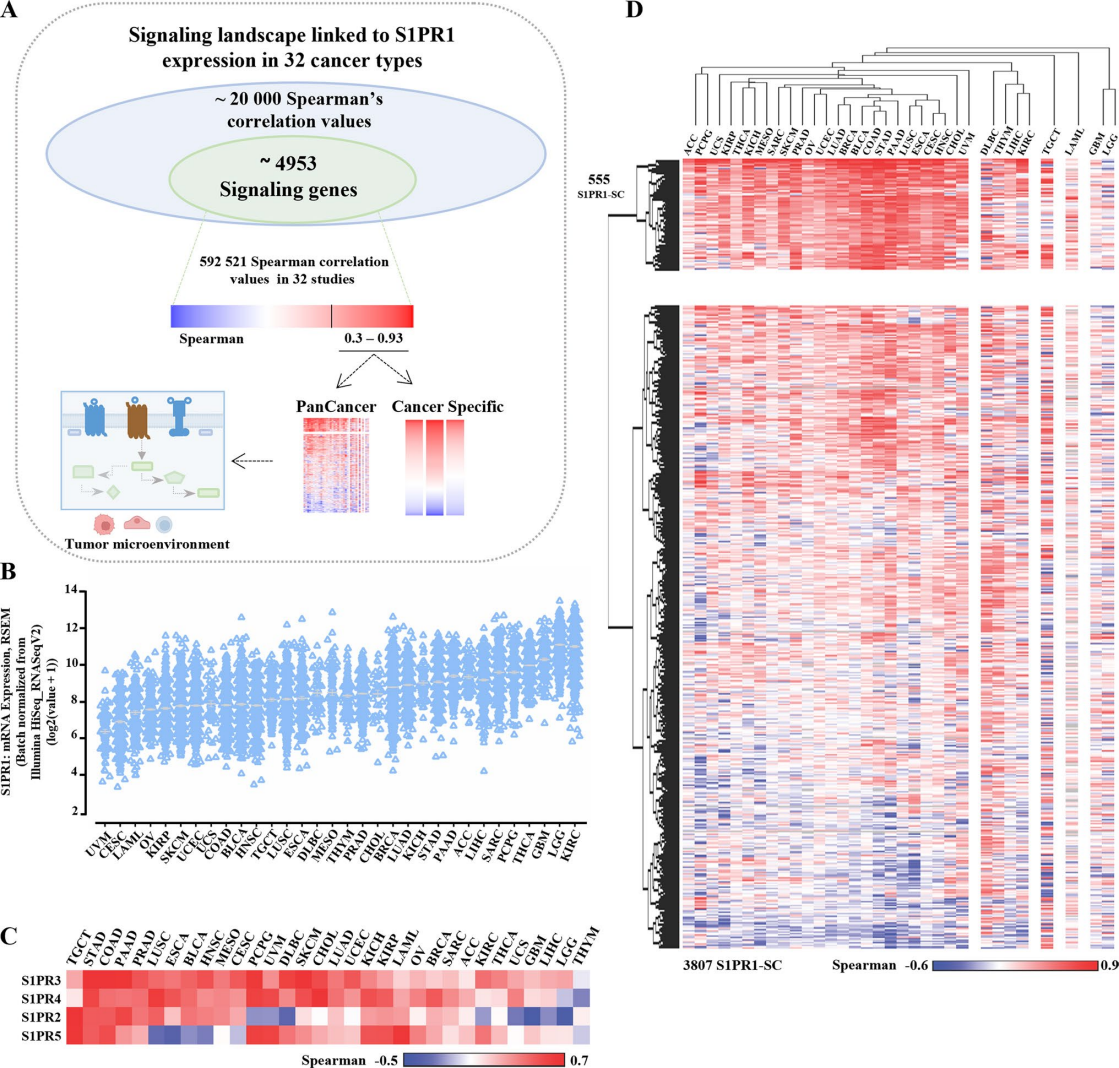
Pan-cancer signaling landscape linked to S1PR1 expression. **A** Data mining strategy focused on cellular signaling hardware oriented to identify S1PR1 signaling companions (S1PR1-SC) in 32 cancer types of the TCGA oncogenomics program. **B** mRNA Expression of S1PR1, RSEM (Batch normalized from Illumina HiSeq_RNASeqV2) (log2(value + 1)). **C** Spearman co-expression patterns of S1PR1 with other S1P receptors. **D** Heatmap illustrating unbiased clustering of 3807 S1PR1-SC in 32 cancer types.

Given the oncogenic impact of S1PR1 signaling in a variety of cancer types and its expression in cancer and stromal cells (Balaji Ragunathrao et al. 2019; Cartier et al. 2020; Deng et al. 2012; Dobersalske et al. 2024; Lee et al. 2010; Lin et al. 2019; Nagahashi et al. 2018; Priceman et al. 2014; Tao et al. 2023a; b), we aimed to identify endothelial and immune pan-cancer S1PR1 signaling partners, looking for those highly coexpressed with this receptor in multiple cancer types, with the ultimate goal to reveal those which together constitute signaling signatures statistically linked to patient survival. With a directory of signaling proteins based on their structural characteristics as described in the SMART platform (Letunic et al. 2021), we tagged the list of transcripts coding for signaling proteins obtained from those with Spearman’s correlation values with S1PR1 of at least 0.3, corresponding to positive significant coexpression with S1PR1 (Fig. 1A). For the analysis of each cancer type, the expression of S1PR1 was consulted in the cBioPortal platform and all the cancer-specific coexpression lists were merged, obtaining data from 10,953 patients and almost 600 000 values from 32 non-redundant TCGA studies (Ding et al. 2018; Hoadley et al. 2018; Sanchez-Vega et al. 2018). S1PR1 transcript was detected in all TCGA cancer types, with a median expression ranging from 6 to 10 RSEM (Fig. 1B). S1PR1 was differentially coexpressed with other S1P receptors in various cancer types, finding only a few examples, such as stomach adenocarcinoma (STAD) and colorectal adenocarcinoma (COAD) where S1PR1 was well coexpressed with all other four S1P receptors (Fig. 1C). Unbiased clustering analysis done with the Euclidian distance algorithm within the Morpheus platform (https://software.broadinstitute.org/morpheus/) led to the initial identification of a pan-cancer group that included 555 S1PR1 signaling companions (S1PR1-SC) common to a cluster including 24 cancer types (Fig. 1D).

### Unbiased clustering of S1PR1-SC in multiple cancer types indicates enrichment of endothelial and immune signaling networks

**Fig. 2:**
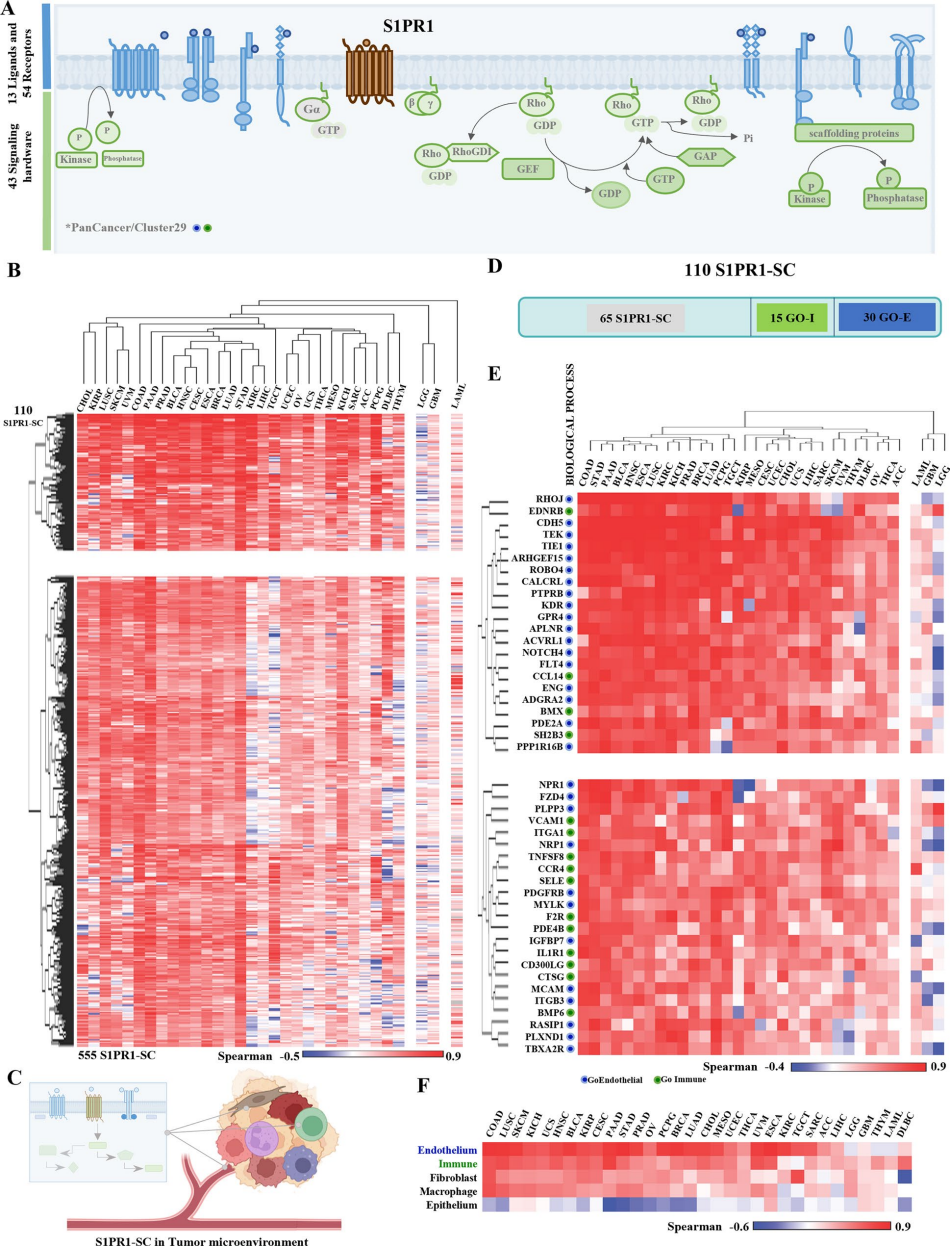
S1PR1-Signaling Companions are found primarily in endothelial and immune cells.** A** Diagram illustrating the expected signaling repertoire linked to S1PR1 expression. **B** Heatmap showing the extended pan-cancer cluster of 110 S1PR1-SC resulting from reanalysis of the initial pan-cancer cluster identified in Fig. 1D (upper branch). **C)** Model showing the expected expression of S1PR1-SC within the tumor microenvironment, including different cell types: endothelial, lymphoid, myeloid, fibroblast and epithelial. **D** Diagram showing the distribution of pan-cancer 110 S1PR1-SC identified as participants of endothelial or immune cellular processes according to Gene Ontology (GO) analysis. **E** Unbiased clustering of endothelial and immmune S1PR1-SC filtered by their corresponding GO biological process. The GO color code is indicated at the bottom. **F** Coexpression of S1PR1 with cellular markers associated with the tumor microenvironment. PECAM1, PTPRC, ITGAM, FAP and EPCAM for endothelial, lymphoid, myeloid, fibroblast and epithelial cell type markers, respectively.

Conceptually, the signaling landscape linked to the oncogenic activities of S1PR1 includes other receptors and their agonists and an intracellular repertoire of signal transducers, effectors and multidomain scaffolding proteins, among others, most of which can be identified based on their structural characteristics. Therefore, tagging the coexpression data with a directory of signaling proteins, based on their structural characteristics, rationalizes the search of pan-cancer signaling companions guided by S1PR1 expression (Fig. 2A). To further select the most highly correlated S1PR1-SC we re-clustered the initial pan-cancer group of 555 transcripts obtaining a group of 110 that were common among a more extended group of cancer types, from 24 in the initial group (Fig. 1D) to 29 in the re-clustered group (Fig. 2B, upper horizontal cluster). Knowing that tumoral S1PR1 signaling occurs in various cells of the tumor microenvironment (Fig. 2C), including endothelial (Balaji Ragunathrao et al. 2019; Cartier et al. 2020), and immune (Deng et al. 2012; Dobersalske et al. 2024; Lee et al. 2010; Priceman et al. 2014), we analyzed the repertoire of 110 S1PR1-SC in the Metascape platform (Zhou et al. 2019), looking to identify those functionally classified as involved in endothelial and immune cellular processes (Fig. 2D). Unbiased clustering of 45 S1PR1-SC, tagged by gene ontology analysis as endothelial or immune (30 and 15, respectively, Fig. 2D), generated two main branches (Fig. 2E, horizontal branches). Only 3 out of 22 transcripts in the upper branch were tagged as immune functional components, whereas the lower branch clustered together the other 12 transcripts tagged as immune (identified with green dots). Most endothelial components (identified with blue dots) were clustered in the upper branch, but the lower branch also included 12 out of 30 endothelial components. Coexpression analysis of S1PR1 with different cell markers (Fig. 2F) confirmed that S1PR1 was mainly coexpressed with markers of tumor stromal cells, particularly endothelial and immune, than with the epithelial cancer cell marker.

### Endothelial repertoire of S1PR1-SC statistically linked to patient survival

**Fig. 3:**
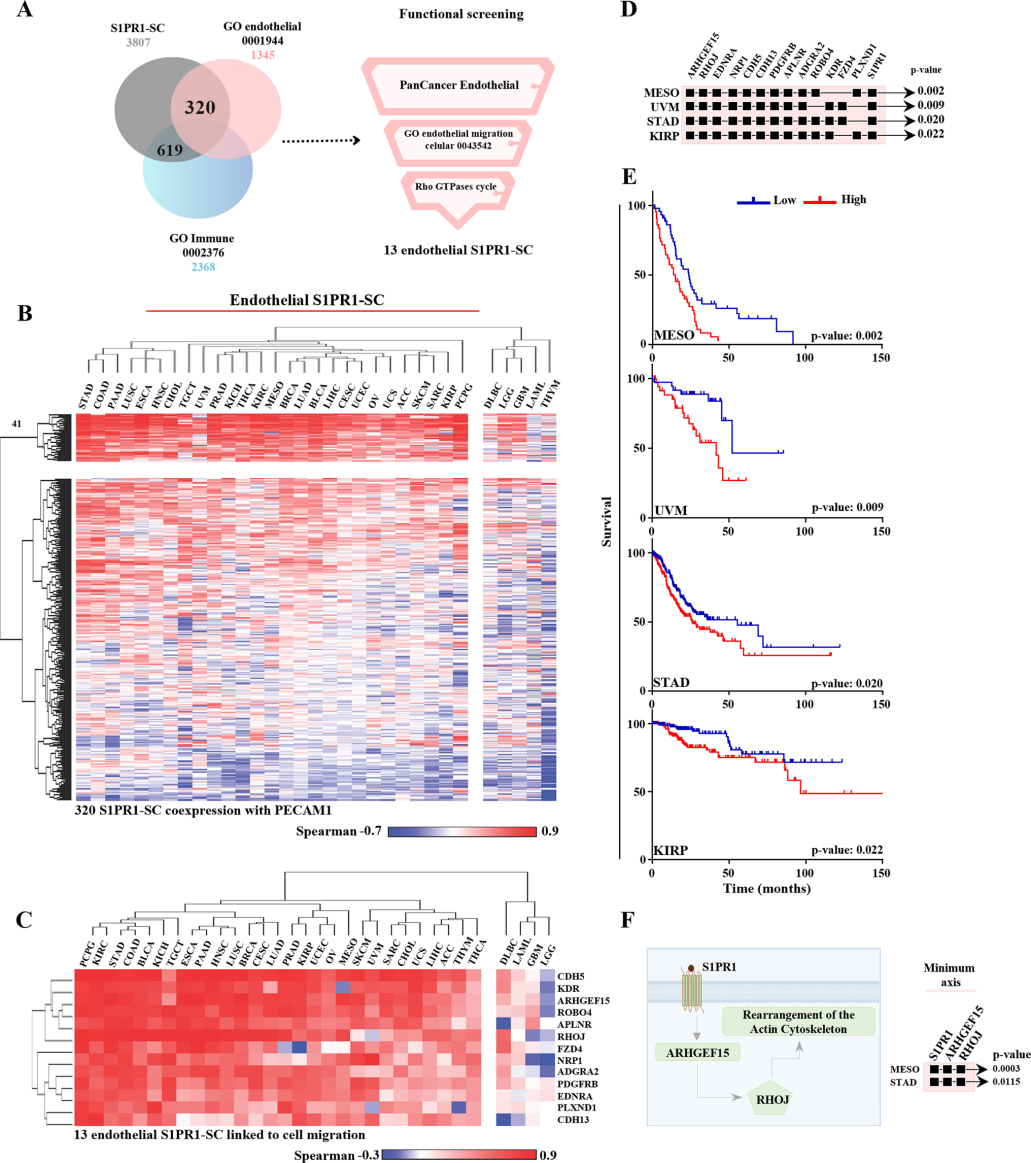
Endothelial S1PR1-SC statistically linked with shorter survival of MESO, UVM, STAD and KIRP cancer patients of the TCGA datasets.** A** Classification of S1PR1-SC as endothelial based on GO biological processes with Spearman ≥ 0.3 (excluding those identified in the immune GO dataset). **B** Coexpression patterns of PECAM1 with the group of endothelial S1PR1-SC, selected by gene ontology analysis, in 32 cancer types resulted in a pan-cancer group of 41 S1PR1-SC. **C** Endothelial S1PR1-SC, confirmed by their coexpression with PECAM1, selected as known participants of cell migration, were analyzed by their coexpression with S1PR1. **D** Endothelial S1PR1-SC, linked to cell migration, that had individual significant correlation with shorter survival (represented by black squares) were analyzed as part of signaling signatures to address potential cumulative risk of shorter patient survival. **E** Survival curves of endothelial pan-cancer S1PR1-SC (shown in** D**) analyzed as transcriptional signatures by multivariate Cox regression conducted in the KM plotter platform. **F** Minimum signaling axis, including S1PR1, one RhoGEF and one Rho GTPase, resulted in a significant cumulative risk of shorter patient survival in MESO and STAD TCGA datasets.

**Fig. 4:**
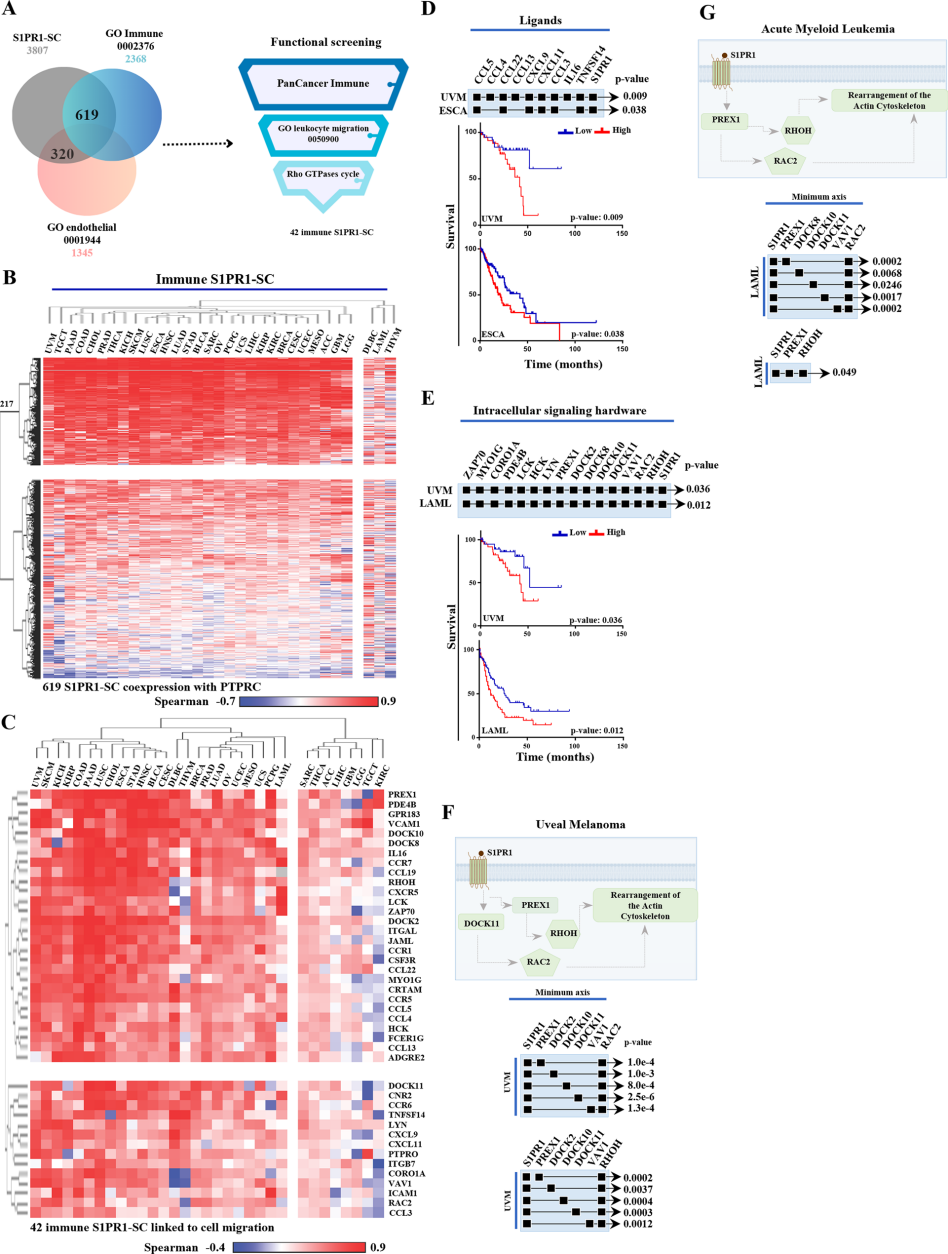
Immune S1PR1-SC statistically linked to shorter survival of ESCA, UVM and LAML cancer patients of the TCGA datasets. **A** Classification of S1PR1-SC as immune based on GO biological processes with Spearman ≥ 0.3 (excluding those identified in the endothelial GO dataset). **B** Coexpression patterns of PTPRC with the group of immune S1PR1-SC, selected by gene ontology analysis, in 32 cancer types resulted in a pan-cancer group of 217 immune S1PR1-SC (upper branch). **C** Immune S1PR1-SC, confirmed by their coexpression with PTPRC, selected as known participants of cell migration, were analyzed by their coexpression with S1PR1. **D–E** Immune S1PR1-SC, known as extracellular ligands **(D)** or components of the intracellular signaling hardware **E** linked to cell migration, that had individual significant correlation with shorter survival (represented by black squares), were analyzed as part of signaling signatures to address potential cumulative risk of shorter patient survival. Survival curves of immune pan-cancer S1PR1-SC were analyzed by multivariate Cox regression conducted in the KM plotter platform. **F–G** Minimum immune signaling axis, including S1PR1, one RhoGEF and one Rho GTPase, resulted in a significant cumulative risk of shorter patient survival in UVM (**F**) and LAML (**G**) TCGA datasets.

Since S1PR1 was coexpressed with endothelial and immune markers and the re-clustered group of 110 S1PR1-SC included about 40% of endothelial and immune functional components (Fig. 2D, based on gene ontology analysis), we decided to apply the endothelial and immune gene ontology criteria to the initial group of S1PR1-SC (containing 3807 transcripts coexpressed with S1PR1 with a Spearman’s correlation value of at least 0.3 in at least one cancer type, as defined in Fig. 1A) to filter those exclusively tagged as endothelial (Fig. 3A) or immune (Fig. 4A) in the Metascape platform (Zhou et al. 2019). We predicted that this approach would reveal cell type enriched signaling repertoires linked to S1PR1 functions in multiple cancers. This strategy led us to identify 320 transcripts which were then subjected to analysis of coexpression with PECAM1 (Fig. 3B), the prototypical endothelial marker, resulting in a pan-cancer group of 41 endothelial S1PR1-SC that was further filtered to select those statistically linked to shorter patient survival and coding for proteins involved in cytoskeletal dynamics and cell migration. This additional selection resulted in a group of 13 endothelial S1PR1-SC (Fig. 3C), all of them structurally recognizable as receptors and members of the signaling hardware related to actin polymerization and cell migration.

To get an initial insight on the potential of endothelial S1PR1-SC as cancer biomarkers, we focused on those whose individual expression was statistically linked to patient survival and analyzed whether, as signaling signatures, were indicative of cumulative risk using the clinical information included in the TCGA studies. As shown in Fig. 3D, 13 endothelial S1PR1-SC were statistically linked to shorter patient survival (marked as black squares) and, analyzed together as signaling signatures (Fig. 3E) were statistically linked to higher risk in mesothelioma (MESO), uveal melanoma (UVM) stomach adenocarcinoma (STAD) and kidney renal papillary cell carcinoma (KIRP), analyzed in the Kaplan Meier Plotter platform (Lanczky and Gyorffy 2021), raising new avenues to investigate the role of S1PR1-driven signaling pathways in these specific cancer types. Given that endothelial functions of S1PR1 involve actin polymerization driven by the activity of RhoGEFs and Rho GTPases (Fig. 3F), we analyzed whether a minimal signaling axis including the S1PR1 receptor, ARHGEF15 and RHOJ was statistically linked to shorter patient survival, which was indeed the case in MESO and STAD (Fig. 3F, right panel).

### Immune repertoire of S1PR1-SC linked to patient survival

Aiming to identify immune S1PR1-SC statistically linked to patient survival, we filtered the initial group of 3807 S1PR1-SC by tagging them with the list of participants of immune processes included in the indicated GO immune functional set (Fig. 4A). We found 619 S1PR1-SC that were identified by gene ontology analysis as participants of immune, but not endothelial, processes. The group of 619 S1PR1-SC, tagged as participants of immune processes, were analyzed in terms of their coexpression with PTPRC, commonly used as an immune marker, resulting in the clustering of a pan-cancer group of 217 immune S1PR1-SC (Fig. 4B, upper cluster). This group was further filtered to select those involved in cell migration and regulation of Rho GTPases resulting in a group of 42 immune S1PR1-SC linked to actin dynamics and cell migration (Fig. 4C), which included chemotactic agonists and receptors, RhoGEFs, Rho GTPases and some tyrosine kinases, among others. All of those whose individual expression was statistically linked to patient survival, indicated as black squares in Fig. 4D–G, were grouped and analyzed as transcriptional signatures organized as ligands (Fig. 4D), intracellular signaling hardware (Fig. 4E) and minimal signaling axis including S1PR1, one RhoGEF and one Rho GTPase, in uveal melanoma (Fig. 4F) and acute myeloid leukemia (Fig. 4G), the cancer types in which the predicted minimal axis were statistically linked to increased risk of shorter survival. Results indicated that transcriptional signatures including various immune pan-cancer S1PR1-SC linked to cytoskeletal reorganization and cell migration were statistically significant in terms of higher risk of shorter survival in cancer patients of the TCGA datasets, particularly UVM, esophageal adenocarcinoma (ESCA) and acute myeloid leukemia (LAML) (Figs. 4D–G).

### Pan-cancer signaling pathways identified from cancer-specific S1PR1-SC

**Fig. 5:**
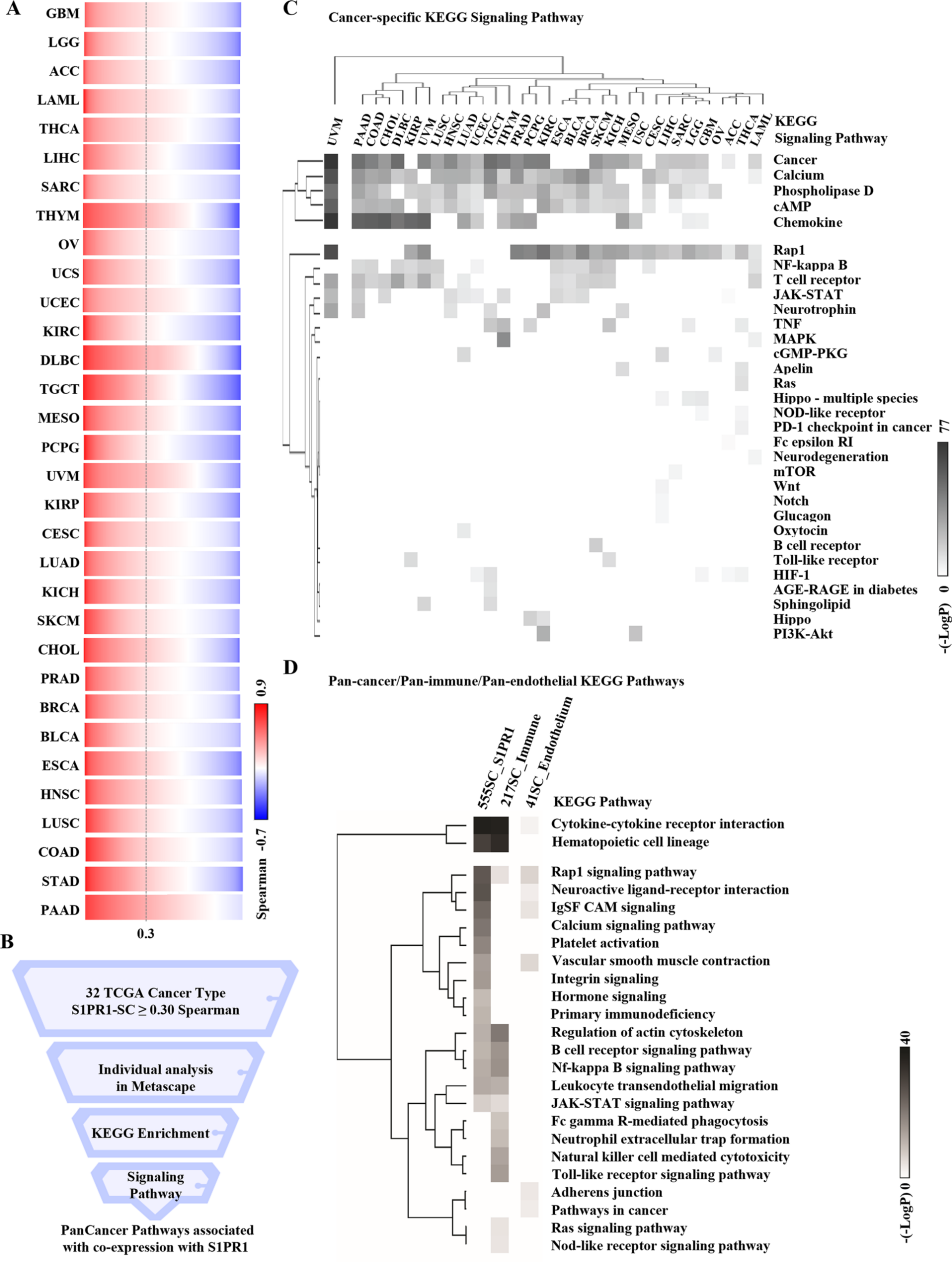
Pan-cancer Signaling Pathways linked to S1PR1 expression.** A** Heatmaps showing individual patterns of gene coexpression with S1PR1 across the 32 cancer types. **B** Graphical representation of the screening strategy of cancer-specific S1PR1-SC to identify pan-cancer signaling pathways linked to S1PR1 expression analyzed at the Metascape platform focusing on canonical KEGG pathways. **C** Unbiased clustering of cancer-specific signaling pathways linked to S1PR1 expression in 32 cancer types. **D** Pan-cancer, pan-immune and pan-endothelial signaling pathways linked to S1PR1 expression. Statistical significance (represented in a grey scale with values shown in the bar at the bottom) was analyzed at the Metascape platform selecting canonical KEGG pathways.

To identify canonical pan-cancer signaling pathways linked to S1PR1 expression, we analyzed each group of cancer type specific S1PR1-SC ordering the list of coexpressed transcripts from highest to lowest Spearman correlation values (Fig. 5A). All those S1PR1-SC with Spearman’s correlation values of at least 0.3 were included in the respective list analyzed in the Metascape platform looking of enrichment of KEGG signaling pathways (Fig. 5B). The individual statistical values were represented together in the heatmap shown in Fig. 5C which highlights that KEGG signaling pathways related to cancer, calcium, phospholipase D, cAMP and chemokine function are common to most cancer types whereas signaling related to Rap GTPases was significant in about 20 cancer types. The highest significant values were found in the list of S1PR1-SC in UVM (Fig. 5C, left column), one of the cancer types with endothelial and immune pan-cancer transcriptional signatures statistically linked to increased risk of shorter patient survival (Figs. 3D–E and 4D–F). In contrast, the pan-cancer, pan-endothelial, and pan-immune S1PR1-SC signatures were associated with distinct, non-overlapping KEGG pathways (Fig. 5D). The main exception was the Rap1 pathway, which also appeared in the cancer-specific gene enrichment analysis (Fig. 5C). This limited overlap likely resulted from the filtering criteria, which selected non-overlapping endothelial and immune S1PR1-SC from GO gene lists. The selection was further restricted by non-biased clustering of pan-cancer endothelial or pan-cancer immune S1PR1-SC based on transcriptional coexpression with PECAM1 or PTPRC, respectively. As a result, functional similarities identified through common KEGG pathways in the cancer-specific analysis (Fig. 5C) were excluded when defining endothelial-specific and immune-specific S1PR1-SC signatures.

**Fig. 6:**
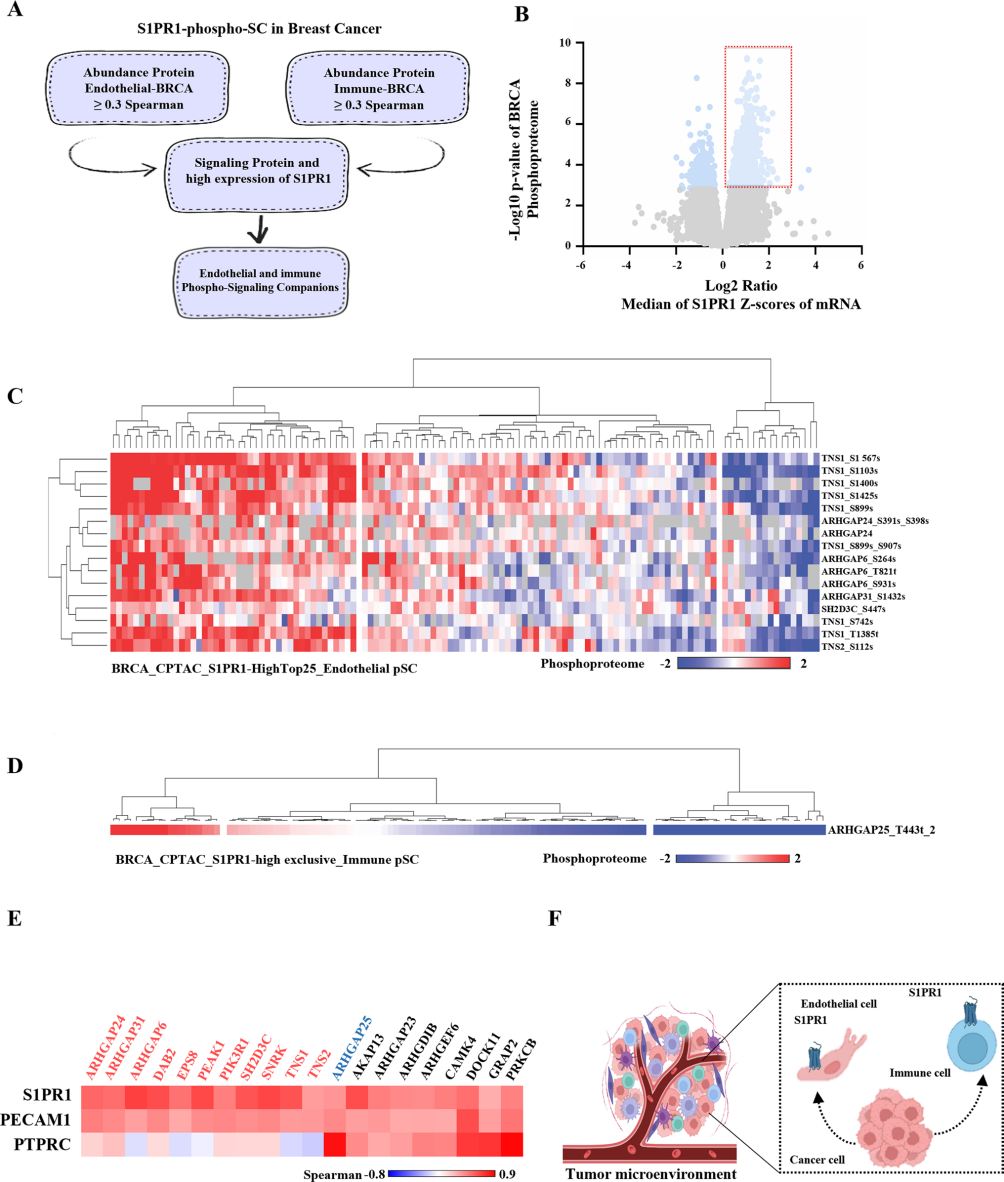
S1PR1-phospho-SC in Breast Cancer. **A** Flowchart describing the parameters used to find endothelial and immune S1PR1-SC within the breast cancer phosphoproteome of the CPTAC dataset. **B** Volcano graph representing the distribution of protein phosphosites in patients divided by their expression of S1PR1 transcript. Selected area represents the phosphosites significantly enriched in patients with high expression of S1PR1. **C** Unbiased clustering of endothelial S1PR1-phospho-SC identified in the breast cancer CPTAC dataset. **D** Heatmap showing the clustering of patients based on the only S1PR1-phospho-SC identified as immune in the breast cancer phosphoproteomic CPTAC dataset. **E** Heatmap showing the co-expression of S1PR1-phospho-SC with S1PR1 and endothelial and immune cellular markers. **F** Schematical representation of S1PR1 in different cells of the tumor microenvironment implying the activation of cell-type specific signaling networks linked to cancer progression

### Phosphoproteomic landscape highly correlated with S1PR1 expression

Given that oncogenic signaling involves phosphorylation-dependent mechanisms that control tumor growth and dissemination (Geffen et al. 2023; Sanchez-Vega et al. 2018), we applied our data mining strategies to the phosphoproteomic dataset of the breast cancer CPTAC study (Krug et al. 2020), looking to identify endothelial and immune S1PR1-phospho-SC (Fig. 6A). Based on S1PR1 expression, we selected the breast cancer phosphosites significantly increased in the group of high S1PR1 expression (Fig. 6B). Unbiased clustering of endothelial S1PR1-phospho-SC (Fig. 6C) segregated a group of patients with phosphorylated TNS1, ARHGAP6, ARHGAP24, ARHGAP31 and SH2D3C, whereas only one immune S1PR1-phospho-SC was identified as the one phosphosite in ARHGAP25 (Fig. 6D). Coexpression with S1PR1 and with endothelial and immune markers of these and other S1PR1-phospho-SC was confirmed at the transcriptomic level (Fig. 6E). Altogether, our analysis indicates the existence of pan-cancer endothelial and immune signaling networks linked to S1PR1 expression within the tumor microenvironment (Fig. 6F).

## Discussion

The role of S1PR1 in cancer progression involves complex networks of communication among endothelial, immune and cancerous cells within the tumor microenvironment leading to chronic signaling that sustains tumor growth and spreading (Balaji Ragunathrao et al. 2019; Deng et al. 2012; Lee et al. 2010; Nagahashi and Miyoshi 2024; Olesch et al. 2022; Priceman et al. 2014; Tao 2023a; 2023b). Mechanistically, it implies particular signaling networks within different cells of the tumor stroma that lead cell–cell communication circuits needed to elicit cancer cell proliferation and metastatic dissemination (Balaji Ragunathrao et al. 2019; Deng et al. 2012; Lee et al. 2010). Signaling cascades activated by S1PR1 in different cancer types and cells involved in tumor-induced angiogenesis, immune cell recruitment and local immunosuppression, facilitating tumor growth and dissemination, certainly involve canonical and still to be identified components such as different G protein coupled receptors and receptor tyrosine kinases, and a complex intracellular signaling hardware of effectors, including kinases, phosphatases, RhoGEFs and GTPases, which we aimed to identify by an unbiased data mining strategy focused on highly coexpressed pan-cancer signaling transcripts. We focused on those that promote dynamic adjustments of the actin cytoskeleton and identified endothelial and immune pan-cancer signaling partners of S1PR1 that individually and grouped as transcriptional signatures were statistically linked to shorter patient survival in various cancer types.

We found little correlation of S1PR1 with epithelial cells. In contrast, this receptor was well correlated with various cells of the tumor stroma, particularly lymphoid and endothelial cells in most cancer types, which is consistent with its role in the immune and vascular systems (Cartier and Hla 2019; Cartier et al. 2020; Hallisey and Schwab 2023; Olesch et al. 2022; Weigel et al. 2023). Various S1PR1-SC have documented roles in oncogenic signaling cascades (Debaugnies et al. 2023; Fukushima et al. 2016; He et al. 2017; Jin et al. 2022; Kim et al. 2014; Komatsu et al. 2022; Saito et al. 2012; Tsutsumi and Ohta 2024), although they have not been studied in the context of S1PR1 signaling, representing potential signaling routes that warrant future investigations. The analysis of accumulated risk indicated that the signaling landscape linked to S1PR1 expression in various cancer types might be clinically significant, pointing to preclinical hypothesis-driven investigations on the role of the signaling companions of S1PR1 in cell migration within the tumor stroma, involving molecular pathways leading to the activation of Rho GTPases. To get an initial insight into the molecular processes linked to the joined action of pan-cancer and cancer specific sets of S1PR1 coexpressed genes, we analyzed Gene Ontology datasets. The analysis pointed to the importance of chemotactic pathways, intracellular second messengers such as calcium and cAMP, and activities of phospholipase D and the small GTPase Rap1 in the oncogenic S1PR1 signaling network. These findings are consistent with the documented role of Rap1 in S1P-induced angiogenesis (Tawa et al. 2010), and lymphocyte trafficking (Ishihara et al. 2020), its regulation by cAMP in the control of endothelial barrier and its dysregulation in inflammatory processes (Schlegel and Waschke 2014) and expand towards the potential role of the identified S1PR1-SC in tumor progression. Besides, some cancer types include S1PR1-SC with more restricted molecular functions such as activation of MAPK, PI3K/mTOR and Hippo pathways, indicating the potential existence of pan-cancer and cancer-type specific signaling networks involving S1PR1 receptors.

Consistent with the documented oncogenic role of S1PR1, known by its effects on tumor stromal cells, our findings contribute to expanding the potential repertoire of markers and targets of precision therapies. Revealing endothelial and immune S1PR1-SC provides useful information to advance on the general idea that promoting tumor vessel normalization and controlling immune cell recruitment contributes to enhance the effects of antioncogenic therapies (Choi and Jung 2023; Guelfi et al. 2024).

### Limitations of the study

Our analysis, based on coexpression data among signaling proteins that statistically correlate with patient survival, identifies molecular components suggestive of potential functional interactions that have to be experimentally tested to sustain mechanistic conclusions.

## Conclusion

Our rational data-mining strategy to characterize the signaling landscape linked to S1PR1 expression, based on the application of multiple filters oriented to select potentially linked signaling elements, guided us to suggest their potential role as part of oncogenic pathways. However, to reach mechanistic conclusions, they have to be experimentally studied to confirm the hypothetical mechanism sustained in our current analysis.

## Data Availability

All data supporting this study are available as public datasets as indicated in the Methods section.
